# KGHC: a knowledge graph for hepatocellular carcinoma

**DOI:** 10.1186/s12911-020-1112-5

**Published:** 2020-07-09

**Authors:** Nan Li, Zhihao Yang, Ling Luo, Lei Wang, Yin Zhang, Hongfei Lin, Jian Wang

**Affiliations:** 1 College of Computer Science and Technology, Dalian University of Technology, Dalian, 116024 China; 2 Beijing Institute of Health Administration and Medical Information, Beijing, 100850 China

**Keywords:** Hepatocellular carcinoma, Information extraction, Knowledge graph

## Abstract

**Background:**

Hepatocellular carcinoma is one of the most general malignant neoplasms in adults with high mortality. Mining relative medical knowledge from rapidly growing text data and integrating it with other existing biomedical resources will provide support to the research on the hepatocellular carcinoma. To this purpose, we constructed a knowledge graph for Hepatocellular Carcinoma (KGHC).

**Methods:**

We propose an approach to build a knowledge graph for hepatocellular carcinoma. Specifically, we first extracted knowledge from structured data and unstructured data. Since the extracted entities may contain some noise, we applied a biomedical information extraction system, named BioIE, to filter the data in KGHC. Then we introduced a fusion method which is used to fuse the extracted data. Finally, we stored the data into the Neo4j which can help researchers analyze the network of hepatocellular carcinoma.

**Results:**

KGHC contains 13,296 triples and provides the knowledge of hepatocellular carcinoma for healthcare professionals, making them free of digging into a large amount of biomedical literatures. This could hopefully improve the efficiency of researches on the hepatocellular carcinoma. KGHC is accessible free for academic research purpose at http://202.118.75.18:18895/browser/.

**Conclusions:**

In this paper, we present a knowledge graph associated with hepatocellular carcinoma, which is constructed with vast amounts of structured and unstructured data. The evaluation results show that the data in KGHC is of high quality.

## Background

Hepatocellular carcinoma is one of the most general malignant neoplasms in adults with high mortality. It accounts for 45% of the world’s deaths and is the most common cause of death in people with cirrhosis [1]. Although the prevention, diagnosis and treatment techniques have been progress, the morbidity and mortality are still on the rise [2, 3]. Therefore, hepatocellular carcinoma has become a hot topic in life science researches and there is a growing trend of using the medical knowledge from the open field. At present, biomedical database is the main source of biomedical information. The majority of biomedical databases are manually extracted and curated by human experts from literatures. Since the amount of biomedical literatures is increasing rapidly, it is difficult for interaction database curators to detect and curate the information efficiently. Therefore, biomedical knowledge usually cannot be updated in time.

Google introduced the concept of knowledge graph in 2012 [4], which aims to better represent unstructured, semi-structured and structured information on the Internet. A knowledge graph is expressed in triples which include object, relation and subject. Compared with biomedical databases, the knowledge update is faster in knowledge graph [5, 6]. As an important vertical application field of knowledge graph, biomedical knowledge graph has already attracted much attention. Yuan et al. constructed a biomedical domain-specific knowledge graph with minimum supervision [7]. Knowlife is a large biomedical database which applies seed facts of 13 relations to extract sentence-level and document-structure patterns for knowledge graph construction and achieved high precision with typing and mutual-exclusion constrains for pruning out invalid candidate facts [8]. In addition, there exist many different types of biomedical knowledge base. For example, SIDER [9] and AMDD [10] contain the information related to drug. Diseasome [11], ParkDB [12], and ChemProt [13] describe disease and disease related gene information.

However, to the best of our knowledge, currently there is not a single, aggregated source about hepatocellular carcinoma available. As a consequence, the healthcare professional has to traverse across several data portals to retrieve relevant knowledge before using it for drug repurposing or diagnosis for hepatocellular carcinoma. This is quite inconvenient for the research of healthcare professionals. Therefore, integrating the knowledge of hepatocellular carcinoma from the database and excavating the knowledge of hepatocellular carcinoma from the large amount of medical literatures is of great significance.

In this paper, we present a knowledge graph for hepatocellular carcinoma. The main contributions of our work can be summarized as follows.

- We constructed a knowledge graph for hepatocellular carcinoma (KGHC) by fusing the data extracted from structured and unstructured sources. And we manually checked the triples to ensure the accuracy of KGHC. The evaluation results show that the data in KGHC has a high quality. This knowledge graph could be an important supplement to existing medical resources for hepatocellular carcinoma.
- To construct KGHC, the knowledge triples need to be from the huge amount of unstructured textual content. Such extraction task is challenging and requires a lot of manual efforts. And this process can be both error-prone and labor-intensive. Therefore, in this paper, we propose an approach to extract the triple from unstructured data automatically.
- Since the extracted entities are usually full of noise, we applied a biomedical information extraction system, named BioIE, to filter the data in KGHC. The experimental results show that BioIE achieves the state-of-the-art result.
- In order to integrate the extracted data from different sources, we propose a fusion method to fuse the extracted data.

## Methods

**Fig. 1:**
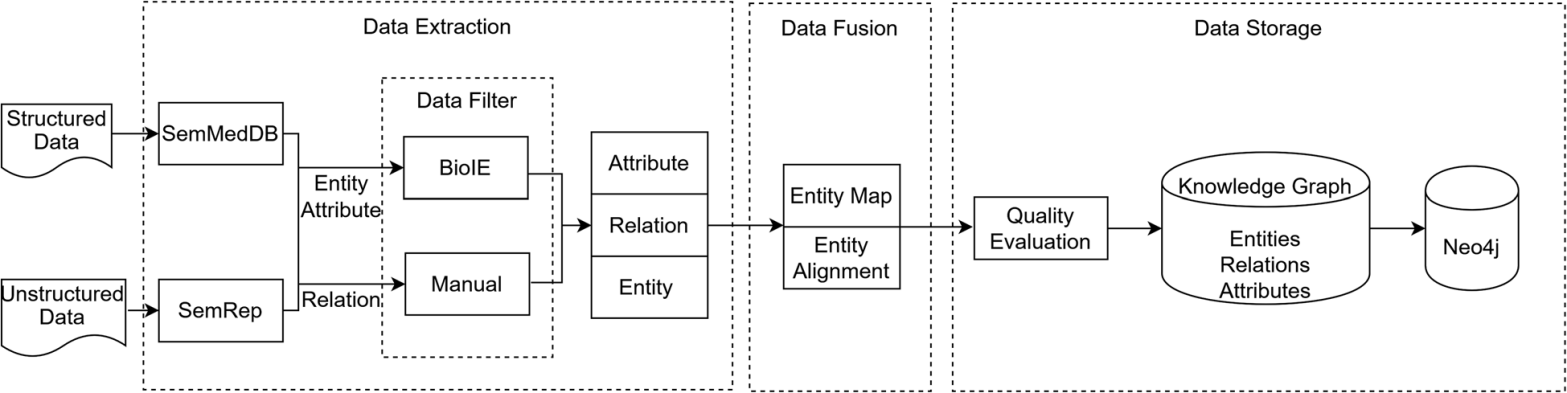
The processing flow of constructing the KGHC

The construction of KGHC mainly includes three parts: data extraction, data fusion, data storage and application. Figure 1 shows the processing flow of our method. Specifically, we first extracted entities, relations and attributes about hepatocellular carcinoma from structured data and unstructured data. Since the extracted entities are always full of noise, we applied a biomedical information extraction system, named BioIE, which used to filter the data in KGHC. Secondly, we proposed a fusion method which is used to fuse the extracted data. Finally, we stored KGHC in Neo4j graph database. The detailed description of our method is presented in the following sections.

### Data schema

The schema of KGHC comes from the Unified Medical Language System (UMLS) [14]. UMLS is a metathesaurus, the largest collection of biomedical dictionaries containing 2.9 million entities and 11.4 million entity names and synonyms [15]. UMLS contains complex taxonomy, including physical objects, events, or even medical equipments. Since our knowledge graph focuses on hepatocellular carcinoma, we only choose parts of UMLS related to our work. By dividing the taxonomy of UMLS, we have summarized nine concepts for our knowledge graph driven by the requirements of analyzing. The concepts of our knowledge graph include: drug, DNA, RNA, gene, protein, cell, disease, phenotypic abnormality and therapeutic-technique. The concept of drug in our knowledge graph include chemical. Then, according to the concepts, we filter the relationship from the UMLS Semantic Network. There are totally 22 relationships in KGHC.

### Data extraction

According to the main source of biomedical information at present, we extracted the data from unstructured data and structured data. In data extraction section, we first used SemRep to extract entities, attributes and relations from unstructured information in literature and Internet. Then, we extracted the triples from structured information in SemMedDB. Finally, we applied BioIE filter out the noise in extracted data. The details are described as follows.

#### Data extraction from unstructured data

The unstructured data contain the latest biomedical information. To keep our knowledge graph current and updated of this ongoing research, we decide to extract knowledge of hepatocellular carcinoma from biomedical literature, medical guideline and clinical trial.

Firstly, we used PubMed (https://www.ncbi.nlm.nih.gov/) to retrieve and download MEDLINE [16] abstracts related to hepatocellular carcinoma. PubMed [17] is an online repository, which contains more than 24 million citations for biomedical literature from MEDLINE and life science journals. MEDLINE is an international database of comprehensive biomedical information created by the National Library of Medicine of the United States. It is the most generally used bibliographic abstract database of foreign literature in the field of biomedicine [18].

Secondly, we downloaded the medical guidelines about hepatocellular carcinoma from UpToDate (https://www.uptodate.com/home). UpToDate is a clinical decision support system based on the principles of evidence-based medicine. It has become the main resource for doctors to acquire medical knowledge during diagnosis and treatment, and provides them with continuous updated information based on the principles of evidence-based medicine.

At last, we obtained the clinical trials about hepatocellular carcinoma from ClinicalTrials.gov by a rule-based method. ClinicalTrials.gov (https://clinicaltrials.gov/) is a resource provided by the National Library of Medicine. It is a worldwide database of funded clinical studies that includes 299,634 studies in 50 countries and 208 cities. There are many unfinished trials in ClinicalTrials.gov. In this work, we only extracted the completed trials.

All these unstructured data do not provide data dumps directly so that we have to extract the entities and relations from these texts. Therefore, we introduce SemRep [19], an information extraction system to extract triples from these unstructured data. SemRep is originally developed for biomedical research and has been extended to the fields of influenza epidemic prevention, health promotion and medical informatics. It is a program base on UMLS that extracts three-part propositions, called semantic predications, from sentences in biomedical text. Predications consist of a subject argument, an object argument, and the relation that binds them [20]. Take the sentence *“Obesity - A number of observational studies have linked excess body fat with a higher risk for HCC.”* as an example*.* From the sentence, the predication *associated_with(Obesity, HCC)* is extracted by SemRep. *Obesity* is the subject, *HCC* is the object and associated_with is the relation between subject and object.

In this paper, we used SemRep extract the entities, relations and attributes (i. e., entity id, text name, entity_start_index, entity_end_index, entity type and source) from the unstructured data. Since it can obtain all the triples that exist in sentence, we only choose parts of triples related to hepatocellualr carcinoma. Table 1 shows the attributes of our knowledge graph.

**Table 1:** The Attributes of Knowledge Graph

Attribute No.	Attribute Name	Remarks
1	PMID	The PubMed abstract ID from which the entity is extracted
2	Text Name	The mention of the entity in sentence
3	Entity ID	The id of the entity
4	Entity Type	The type of entity
5	Entity_Start_Index	The first character position (in sentence) of the text denoting the entity
6	Entity_End_Index	The last character position (in sentence) of the text denoting the entity
7	Source	The source of triple (e.g., UptoDate)
8	Sentence	The sentence including the triple

#### Data extraction from structured data

Biomedical database is the main source of biomedical information which contains a lot of biomedical knowledge related to hepatocellular carcinoma. The National Center for Biotechnology Information (NCBI) portal [20] exposes various biological databases, such as the GenBank nucleic acid sequence database [21, 22] and the BioProject database [23], and also provides tools for retrieval and analysis of the data. In this work, we obtained knowledge about hepatocellular carcinoma in SemMedDB. SemMedDB [24] contains the data which SemRep extracted from MEDLINE abstracts, and includes 96 million relational prediction databases [25, 26]. We extracted data about hepatocellular carcinoma from SemMedDB, and then filtered the data according to the ontology.

In process of data extraction, we found that some information extracted from SemMedDB appears in the sentences that are not conclusive. For example, from the sentence *“Is excessive alcohol abuse one of the causes of hepatocellular carcinoma?”*, SemMedDB extracts the triple *cause (alcohol, hepatocellular carcinoma)*. However, this triple maybe not accurate since it is extracted from a question sentence. In order to solve this problem, we use a rule-based method to filter the data to ensure the accuracy of the triple. And we retain the attributes shown in Table 1. Specifically, if the sentence is a question sentence, we delete this sentence.

### Data filter

The entities which extracted by SemRep may contain some noise. Taking the extracted entities are incomplete as an instance, it always affects the quality of the knowledge graph. For example, B7–1 gene is recognized as gene, and quinone reductase (QR) induction is recognized as induction. To ensure the high-quality of the data in our built knowledge graph, we applied BioIE to automatically extract the multi-type entities from biomedical literature (such as disease, drug, protein, gene, DNA, RNA and cell).

**Fig. 2:**
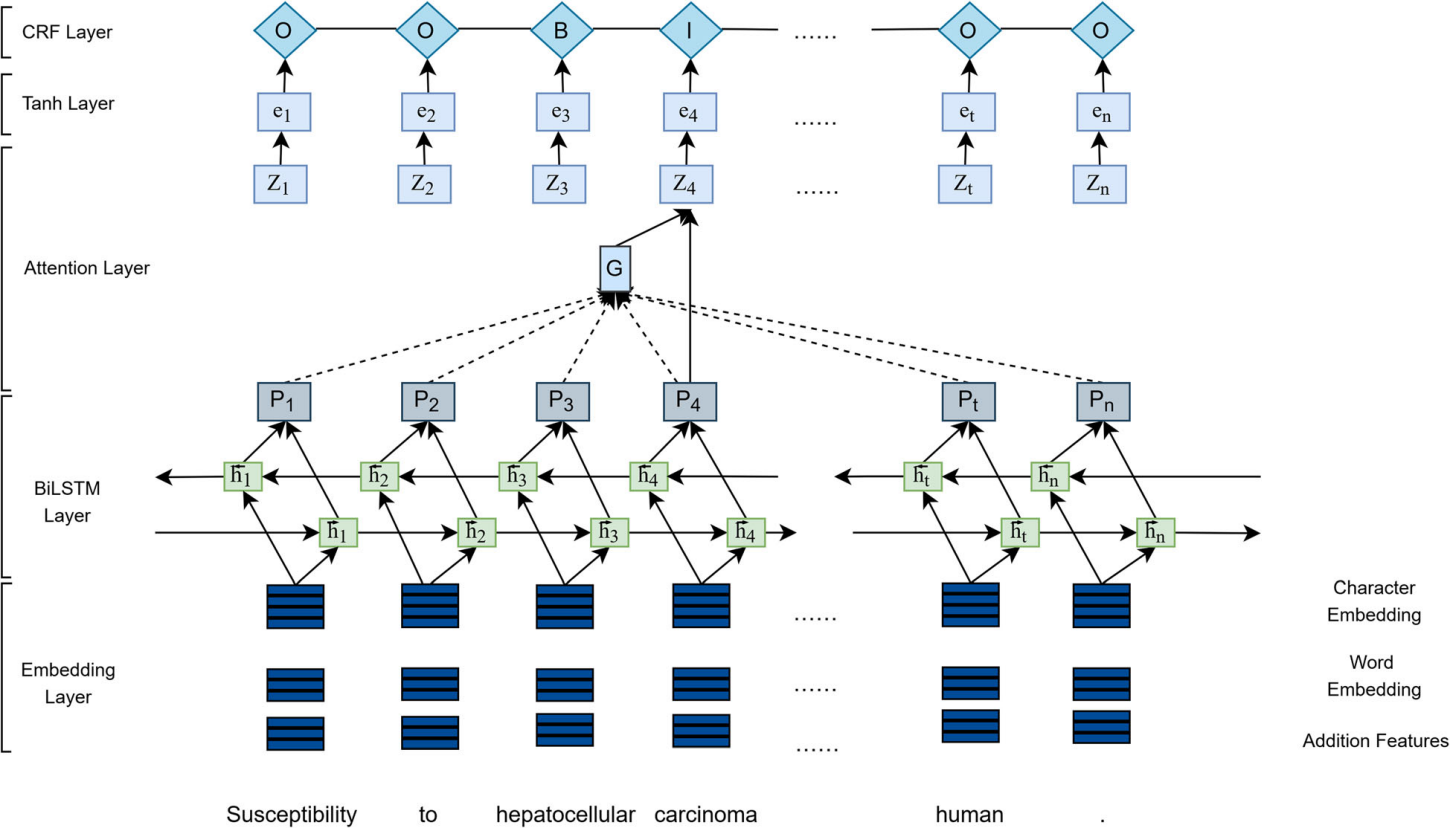
The architecture of Att-BiLSTM-CRF

We proposed the Attention-based named entity recognition model (Att-BiLSTM-CRF) [27] and used it in BioIE. Compared with the traditional BiLSTM-CRF [28] model, Att-BiLSTM-CRF can solve the problem of the inconsistent labels. Attention mechanism in the model is used to learn contextualized embedding, and it can ensure the consistency of entity label and the accuracy of entity recognition. Figure 2 shows the architecture of the Att-BiLSTM-CRF model. A document *D* = (*X*_1_, …, *X*_*t*_, …, *X*_*m*_) containing *m* sentences as an input, and each sentence is expressed as (*x*_1_, …, *x*_*t*_, …, *x*_*n*_), where *n* is the number of the words in sentence [29]. The first layer of the model is the embedding layer, which the concatenation of the character embedding, word embedding and addition features (i. e., POS information and chunking information, et al.) as input is fed into the BiLSTM layer. The BiLSTM layer is used to extract sentence features automatically. It is consisted of a forward LSTM which computes a representation ${\overrightarrow{h}}_t$ of the sequence from left to right, and a backward LSTM which computes a representation ${\overleftarrow{h}}_t$ of the same sequence in reverse [30]. And the concatenation of ${h}_t=\left[{\overrightarrow{h}}_t;{\overleftarrow{h}}_t\right]$ is the output of the BiLSTM layer.

In attention layer, we apply the attention mechanism to focuses on the related tokens in the different sentences of a document to address the tagging inconsistency problem. Specifically, the attention layer is used to capture similar word attention at the document-level. The attention matrix *A* is used to calculate the similarity between the current target word and all words in the document. The attention matrix *A*, which can be described as *a*_*i*, *j*_, can be computed by formula (1).

1 $$ {a}_{i,j}=\frac{\exp \left( score\left({x}_i,{x}_j\right)\right)}{\sum_{k=1}\exp \left( score\left({x}_i,{x}_k\right)\right)} $$

The similarity between *x*_*i*_ and *x*_*j*_ can be calculated by the following four alternatives, (i.e. manhattan distance, euclildean distance, cosine distance and perceptron), where *W* is a weight matrix [27].

2 $$ score\left({x}_i,{x}_j\right)=\left\{\begin{array}{c}W\mid {x}_i-{x}_j\mid \\ {}W{\left({x}_i-{x}_j\right)}^T\left({x}_i-{x}_j\right)\\ {}\frac{\frac{W\left({x}_i\bullet {x}_j\right)}{\mid {x}_i\Big\Vert {x}_j\mid }}{\tanh \left(W\left[{x}_i;{x}_j\right]\right)}\end{array}\right. $$

Then formula (3) is used to calculate a document-level global vector *G*, where H is the output of the BiLSTM layer.

3 $$ G= AH $$

Next, to predict confidence scores for the word, a tanh layer is constructed on top of the attention layer. At last, instead of decoding each label independently, the CRF layer is added to decode the best tag path in all possible tag paths. We trained the model on the dataset of CDR and NLPBA. And to verify the effectiveness of the model, we selected a single category (chemical compound) recognition on the CHEMDNER dataset provided by BioCreative IV for comparative experiments. As shown in Table 2, our method achieves an F-score of 90.84% with no addition feature engineering, which is a state-of-the-art result.

**Table 2:** The result of Att-BiLSTM-CRF model on CHEMDNER dataset of BioCreative IV

Method	Precision(%)	Recall(%)	F-score(%)
tmChem [31]	89.09	85.75	87.39
Lu et al. [32]	88.73	87.41	88.06
RNNA-CRF [33]	91.14	88.27	89.68
BiLSTM-CRF [28]	91.31	87.73	89.48
Att-BiLSTM-CRF	91.65	90.04	90.84

**Fig. 3:**
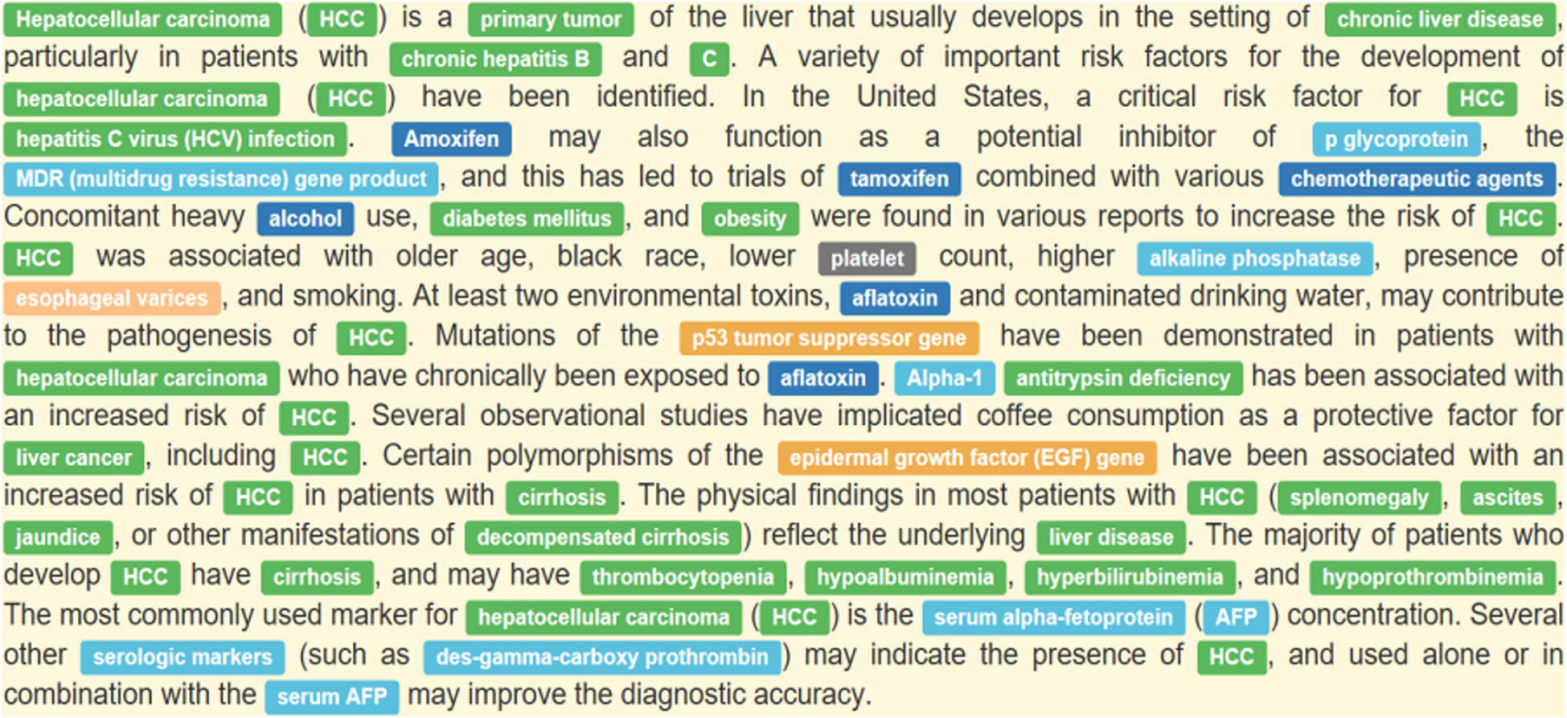
An example of BioIE output

In this work, BioIE is used to extract the entities and attributes of hepatocellular carcinoma from structured data and unstructured data. Specifically, given a sentence which has been extracted the triples from structure data and unstructured data, BioIE extracts the entities and attributes (as shown in Table 1) from this sentence. Figure 3 shows an example of BioIE. However, different data extraction method extracts different information. Take the sentence “*Combined modality doxorubicin-based chemotherapy and chitosan-mediated p53 gene therapy using double-walled microspheres for treatment of human hepatocellular carcinoma.”* as example, BioIE extracts the entities *p53 gene* and *hepatocellular carcinoma* and SemRep extracts the triples *associated_with*(*chemotherapy*, *hepatocellular carcinoma)* and *associated_with*(*gene*, *hepatocellular carcinoma)* from sentence. So it is important to align entities and triples between BioIE and SemRep. Therefore, we proposed a rule-based filter method.

We used SemRep and BioIE to extract the entities and attributes from the same sentence. If the text names of entities in triple are extracted by both SemRep and BioIE, and the attributes of entities (including text name, entity_start_index, entity_end_index, sentence and source) are the same, this triple is retained. Otherwise, it is removed. For example, in the above sentence, SemRep extracts the entities *gene* and *hepatocellular carcinoma* and BioIE extracts the entities *p53 gene* and *hepatocellular carcinoma*. The attributes of entity are different. So we removed the triples *associated_with (gene*, *hepatocellular carcinoma).* The entity filter method can filter the extracted data in KGHC. Corresponding, in order to ensure the accuracy of the extracted relationship, we manually filter the relations between the entities after using BioIE to filter the data.

### Data fusion

The data which we extracted from structured and unstructured data have some noise, such as redundant, complementary, and sometimes have conflicts on some values. To ensure the accuracy of the data in the knowledge graph, we fused the data in two steps: entity mapping and entity alignment. For entity mapping, the same entity has different entity names or the same entity name represents different substances. For example, both *HCC* and *Hepatocellular Carcinoma* denote the disease *hepatocellular carcinoma*. In this work, we extracted the standard name and text name of the entity from SemRep and BioIE (the text name is the mention of the entity in sentence and the entity standard name is the preferred name of the entity). We use the standard name of entity as the entity name, and use the text name as an attribute of the entity to map entity. Take the sentence “*Alcohol can cause HCC”* as an example. *HCC* is the text name and *Hepatocellular Carcinoma* is the standard name. We use *Hepatocellular Carcinoma* as entity name and use *HCC* as an attribute of the entity.

For entity alignment, in data filter, we used BioIE to filter the entities and attributes. The entity name (standard name) and attributes (entity type) extracted by SemRep and BioIE may be different. For example, in the above sentence, the entity name of *HCC* is *Primary carcinoma of the liver cells* in SemRep and *Hepatocellular Carcinoma* in BioIE. We use the JaccardSimilarity [34] to calculate the similarity of entity name between BioIE and SemRep. Only when the value of JaccardSimilarity is 1, is the triple retained. Otherwise, we manually checked the consistency of entity names. We adopted a voting strategy to solve the inconsistencies (e.g. the ones of entity type), i.e., for a given entity, we tend to trust the type which has the most support.

### Data storage and application

The main storage forms of knowledge graph include Resource Description Framework (RDF) and graph database. RDF can establish links between data and query [35] through Sparql. Graph database which can store entities and relations of knowledge graph in the form of graph. Compared with the RDF, the graph database has a better readability. Neo4j is a graph database which can query and update data by using graph query language Cypher. And it provides REST structure, which can be integrated into environments based on PHP, NET, Python and JavaScript [36].

**Fig. 4:**
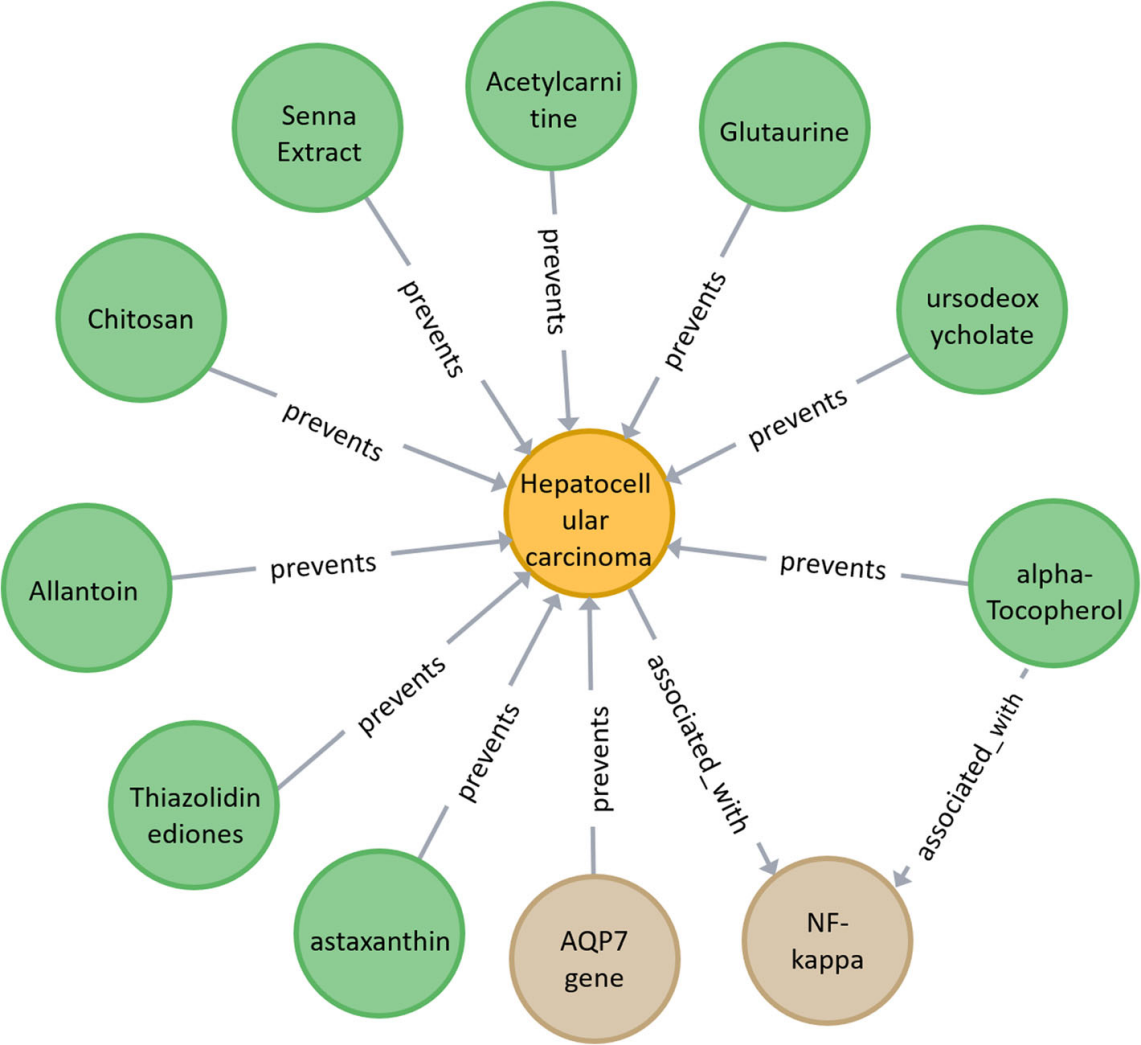
A partial display of KGHC

In this work, Neo4j is used to store the data. We imported the triples into Neo4j through the Neo4j-import tool. Figure 4 is a partial display of KGHC. When KGHC is opened in Neo4j, a network is displayed, in which the nodes refer to the entities and the edges refer to the relations between the entities. The biomedical researchers can use Cypher to search the entities and relations.

In data extraction section, we obtained a two-level relation knowledge graph. For example, *hepatocellular carcinoma* has a relationship with *Hepatitis A*. We extracted data from the structure data and unstructured data that is related to *Hepatitis A* and find that *Glucagon* has a relationship with *Hepatitis A*. So the Glucagon may be related to *hepatocellular carcinoma*. It may help biomedical researchers discover the substances related to *hepatocellular carcinoma*.

**Fig. 5:**
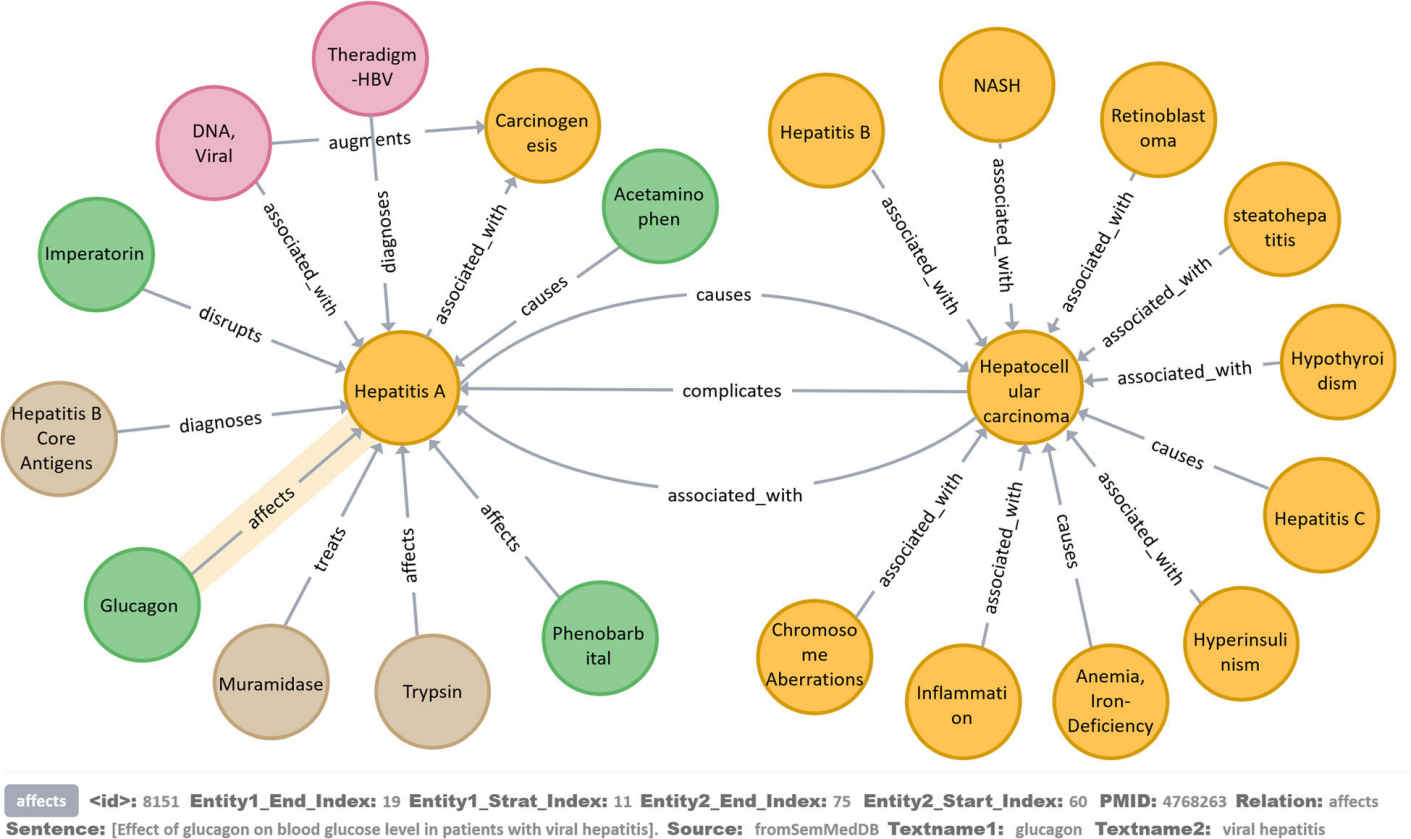
Parts of network between hepatocellular carcinoma and Hepatitis A

In addition, KGHC also contains a large number of attribute information. Selecting a node or edge in the network, users can see the detailed information of attributes about the triple at the bottom of the interface, as shown in Fig. 5. The detailed description of attribute is shown in Table 1. When biomedical researchers propose research hypotheses, they can obtain relevant research articles through the PMID provided by KGHC. In our opinion, KGHC can support the analysis of the *hepatocellular carcinoma* network and may facilitate the discovery of the molecular mechanisms behind the it.

## Results

### Overview of knowledge graph

**Fig. 6:**
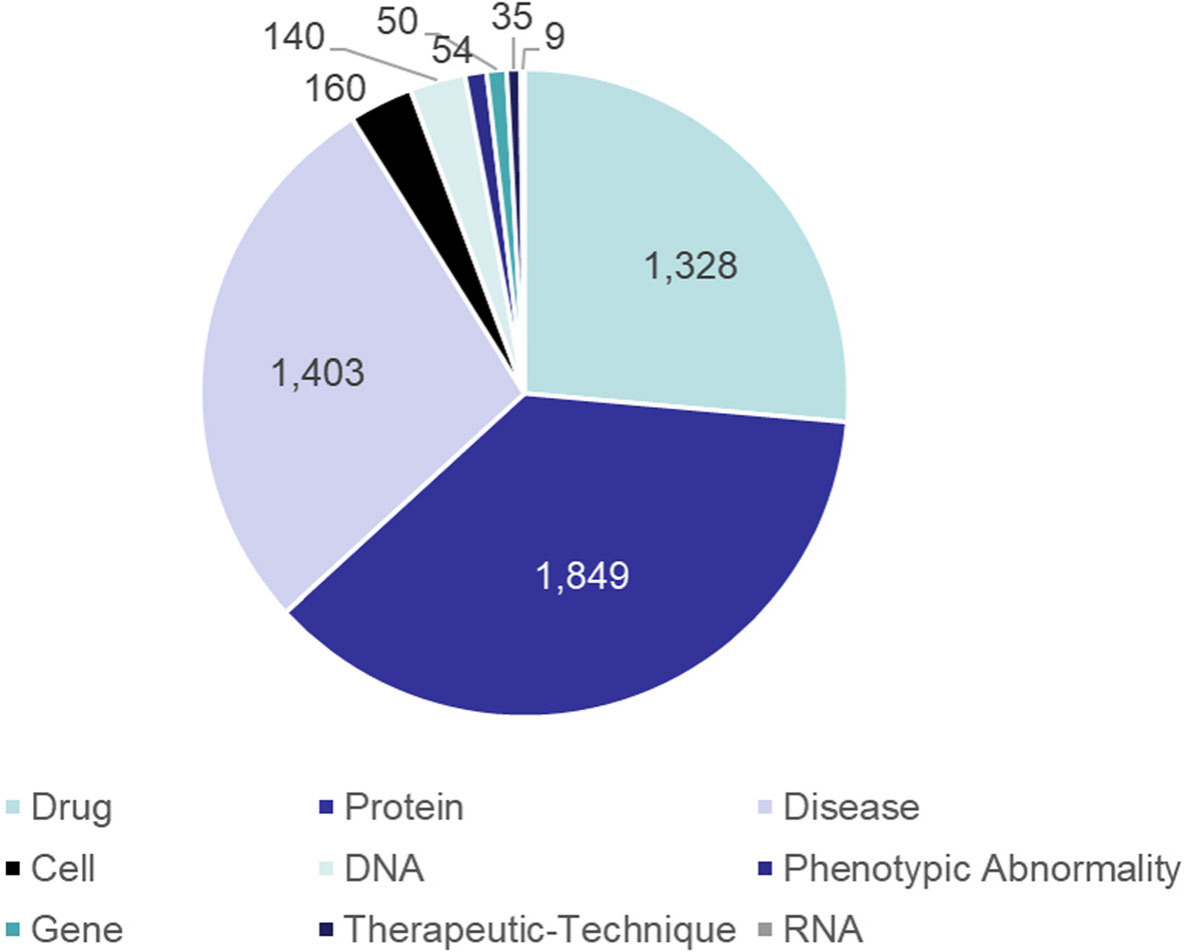
Data distribution in different categories of knowledge graph

KGHC is stored in the form of triples. It has 5028 entities and 13,296 triples. Specifically, there are 1328 drugs, 1849 proteins, 1403 diseases, 160 cells, 140 DNAs, 54 phenotypic abnormalities, 50 genes, 35 therapeutic-techniques and 9 RNAs (as shown in Fig. 6).

**Fig. 7:**
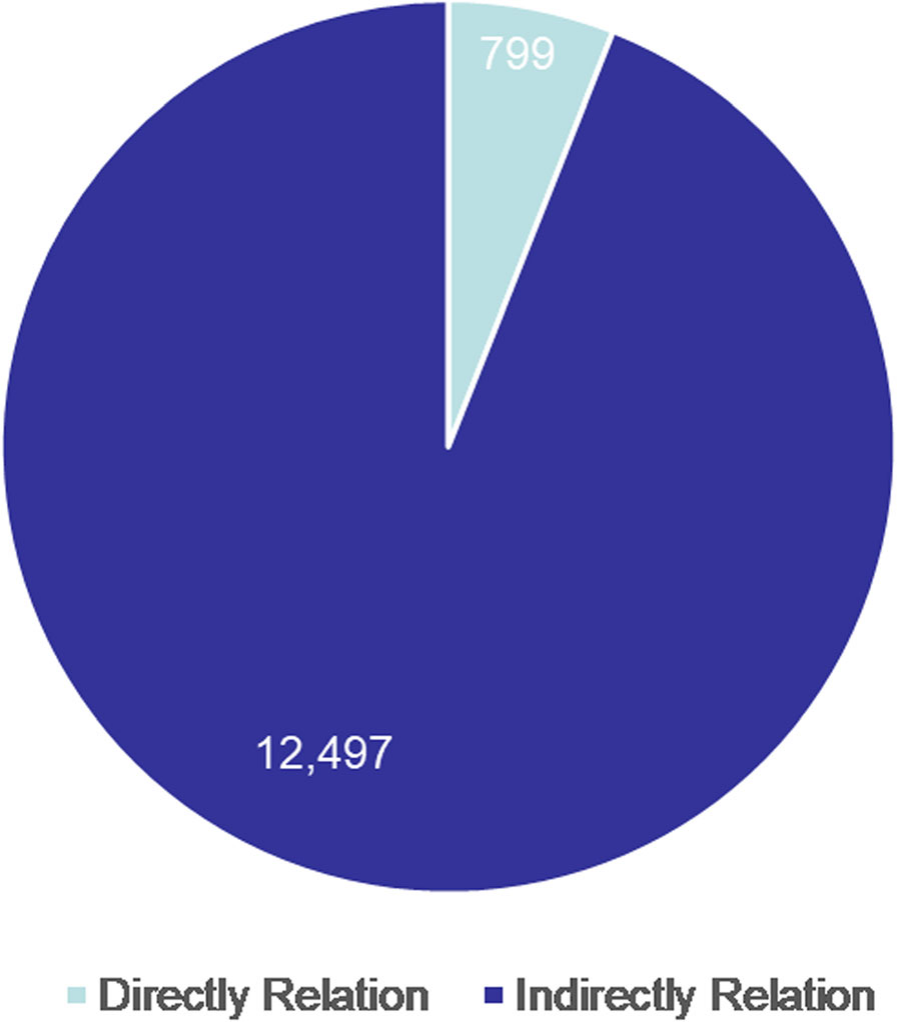
Directly relation and indirectly relation

**Fig. 8:**
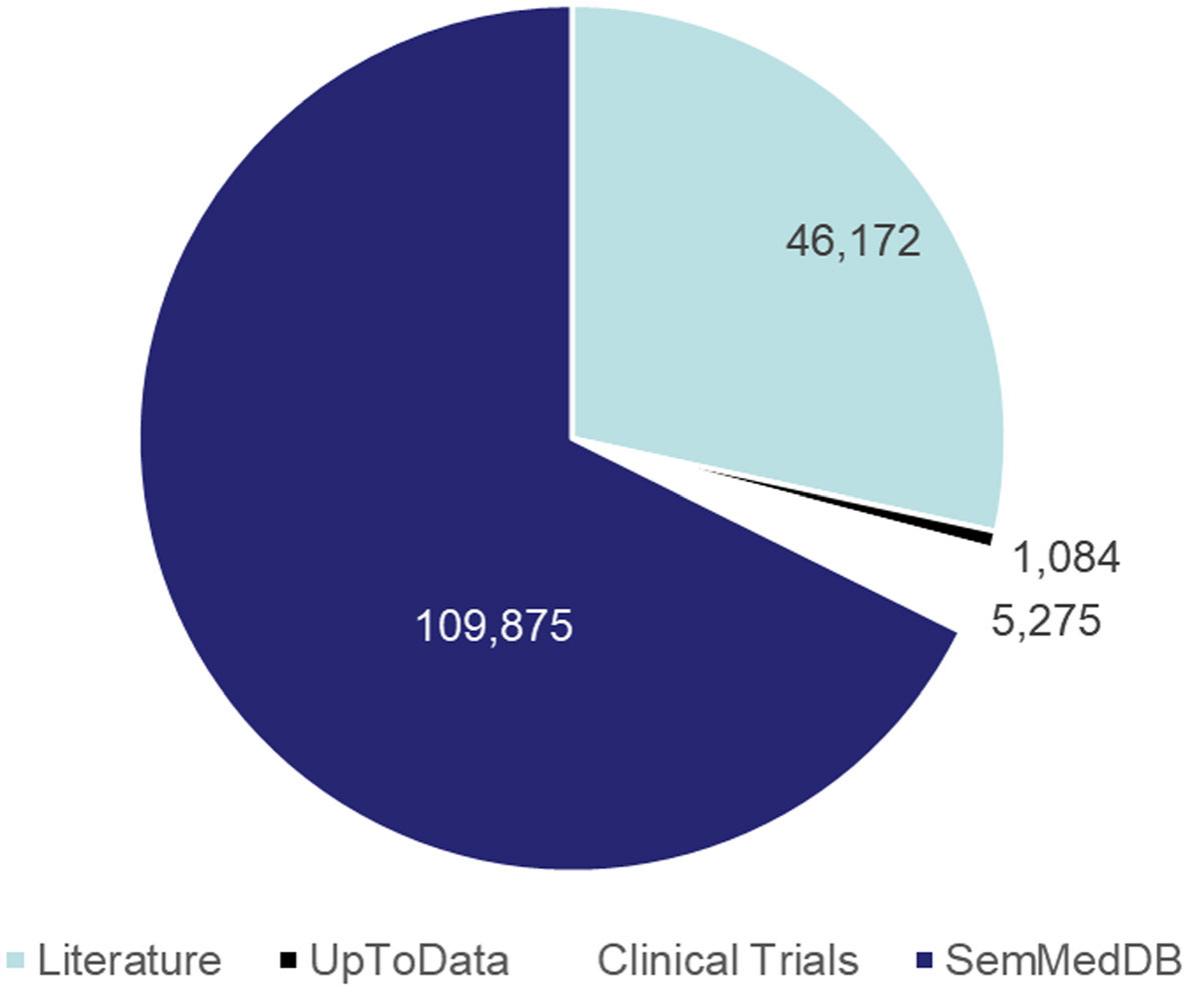
The input corpus of knowledge graph

In addition, KGHC contains 799 triples directly related to *hepatocellular carcinoma*, and 12,497 triples indirectly related to it. The direct relation contains 682 entities, and the indirect relation contains 4726 entities as shown in Fig. 7. The researchers can use the direct and indirect relations to propose new hypotheses. Figure 8 shows the input source of the knowledge graph. It contains four parts: 46,172 sentences of literature, 1084 sentences of UpToDate, 5275 sentences of ClinicalTrials.gov and 109,875 sentences of SemMedDB. Through analyzing the data, we found the following facts,

- The number of the sentences of various sources is 162,406. It is far bigger than the number of the triples in our knowledge graph (13,296), which shows the large-scale redundant information exists between different data sources. KGHC can help researchers filter out the redundant information, and improve research efficiency.
- KGHC contains 13,296 triples and the number of entities is 5028. That means that an entity may be related to multiple different entities. It is useful for researchers to analyze the relations between different entities and facilitate the discovery of the molecular mechanisms or the treatment method of *hepatocellular carcinoma*.

### Data evaluation

For KGHC, its data quality is of great importance. However, there is no *hepatocellular carcinoma* gold set currently. In data filter process, we filtered the entities and attributes by BioIE and checked the relation manually. Therefore, to assess the consistency of the entities and relations, we measured the pairwise agreement of duplicate annotations using the Jaccard score [37].

If we defined A as the set of annotations of team A, B as the set of annotations of team B, then the Jaccard agreement score could be calculated by counting the number of agreements and disagreements. If a triple relation is true, it is counted as a case of agreement. Take *“Alcohol can cause hepatocellular carcinoma.”* as an example. The extracted triple is *cause* (*alcohol*, *hepatocellular carcinoma*). If one annotator annotates this triple true, another annotates it false, then that would count as a case of disagreement. Formula (4) shows the formula of Jaccard score. We used the simple random sampling method to draw 1000 triples from our knowledge graph to calculate the consistency of the triples (i.e., entity and relation) manually. The accuracy ratio is 81.20%.

4 $$ {S}_{\mathrm{A},B}=\frac{\mid A\cap B\mid }{\mid A\cup B\mid } $$

## Discussion

We analyzed the causes of disagreement for 188 facts from the consistency annotation. As shown in Table 3, we categorized the disagreements as follows:

- Entity recognition: Some entities are not correctly recognized. For example, complex entities are composed of multiple simple entities and special symbols (e. g., *TGF-beta receptor-2* is recognized as *TGF-beta receptor*).
- Entity disambiguation: Obtaining the wrong type of entity caused this error. We align entity types with a voting method, i.e., the entity type receiving the most votes wins. If two types have the same top votes, we will judge manually. Perhaps the wrong type was chosen at the time of voting.
- Inaccurate relation: There is a relationship between the entities, but the extracted relationship is inaccurate. Take “*Phase II trial of amsacrine in patients with hepatoma: a Cancer and Leukemia Group B study*” as example, the extracted triple is *treats (Amsacrine, hepatoma).* However, this triple may be not accurate since the entities may have the relation *associated_with*, but we cannot judge the entities have the relation *treats* in this sentence.
- Non-existent relation: Two entities might merely co-occur within the same sentence without really sharing a relation. When such a triple is extracted, it will result in a false relation.
- Passive relation: Failure to accurately identify passive relationships. For example, an triple *cause (hepatocellular carcinoma, alcohol)* may be extracted from the sentence *“A major risk factor for human hepatocellular carcinoma is alcohol”.* However, this triple may is not accurate since *hepatocellular carcinoma* cannot cause *alcohol*.
- Negation Relation: This kind of error is caused because the negation expression in the text is not detected. For example, from the sentence “*It is disputed whether the growth hormone receptor is present in human hepatocellular carcinoma”,* the extracted triple is *associated_with(growth hormone receptor, hepatocellular carcinoma).* However, we cannot confirm the relation between the entities only according to this sentence.

**Table 3:** Disagreement analysis

Cause of error	Percentage	Percentage based on text genre			
		literature	UpToDate	ClinicalTrials.gov	SemMedDB
Entity Recognition	6.91%(13)	23.08%(3)	30.77%(4)	7.69%(1)	38.46%(5)
Entity Disambiguation	7.44%(14)	28.57%(4)	7.14%(1)	7.14%(1)	57.14%(8)
Nonexistent Relation	16.48%(31)	16.13%(5)	0(0)	6.45%(2)	77.42%(24)
Inaccurate Relation	47.34%(89)	16.85%(15)	2.25%(2)	2.25%(2)	78.65%(70)
Passive Relation	10.63%(20)	20%(4)	5%(1)	0(0)	75%(15)
Negation Relation	11.17%(21)	19.05%(4)	0(0)	0(0)	80.95%(17)

As shown in Table 3, the errors of relationship accounted for about 85% of all errors, and most of them (47.34%) belong to the class of *Inaccurate Relation*.

## Conclusions

In this paper, we present a knowledge graph (KGHC) for hepatocellular carcinoma, which is constructed with vast amounts of structured and unstructured data. We first extracted the entities and relations from the different sources. Then we applied BioIE to filter the data of KGHC. After that, we proposed a method to fuse the extracted data. Finally, we stored the data in the Neo4j which can help researchers analyze the network of hepatocellular carcinoma. In addition, we checked the data manually to ensure the accuracy in KGHC. The evaluation results show that the data in KGHC is of a high quality. KGHC is accessible free for academic research purposes at http://202.118.75.18:18895/browser/. To keep the data in knowledge graph up to date, we plan to update KGHC every six months.

## Data Availability

KGHC is freely accessible at http://202.118.75.18:18895/browser. BioIE is freely accessible at http://202.118.75.18:8893/precision_medicine/index.html.

## Abbreviations

KGHC: Knowledge Graph for Hepatocellular Carcinoma
UMLS: Unified Medical Language System
NCBI: National Center for Biotechnology Information
RDF: Resource Description Framework
